# Intracellular survival of *Staphylococcus aureus* in macrophages during osteomyelitis

**DOI:** 10.1080/21505594.2025.2553789

**Published:** 2025-09-01

**Authors:** William A. Lathram, Christopher D. Radka

**Affiliations:** Department of Microbiology, Immunology and Molecular Genetics, University of Kentucky, Lexington, KY, USA

**Keywords:** *Staphylococcus aureus*, macrophage polarization, osteomyelitis, phagocytosis, intracellular infection, pathogenesis

## Abstract

*Staphylococcus aureus*, traditionally viewed as an extracellular pathogen, is increasingly recognized for its ability to persist intracellularly, particularly within macrophages. This intracellular lifestyle is central to osteomyelitis, a chronic bone infection characterized by persistent inflammation, bone destruction, and impaired repair. Within bone, *S. aureus* exploits macrophage plasticity by driving a shift from pro-inflammatory, bactericidal M1-like states to anti-inflammatory, tissue-reparative M2-like phenotypes. This polarization suppresses immune clearance and promotes an environment conducive to bacterial survival and dissemination. Additional strategies – including biofilm formation, small colony variants, and inhibition of phagolysosomal killing – further enhance persistence and immune evasion. While these mechanisms are well studied in extracellular infections, their role in intracellular survival is increasingly evident. This review synthesizes emerging insights into how *S. aureus* manipulates macrophage function to establish chronic bone infection and highlights therapeutic opportunities targeting macrophage polarization to improve immune-mediated clearance and bone repair in osteomyelitis.

## GRAPHICAL ABSTRACT

**Figure:**
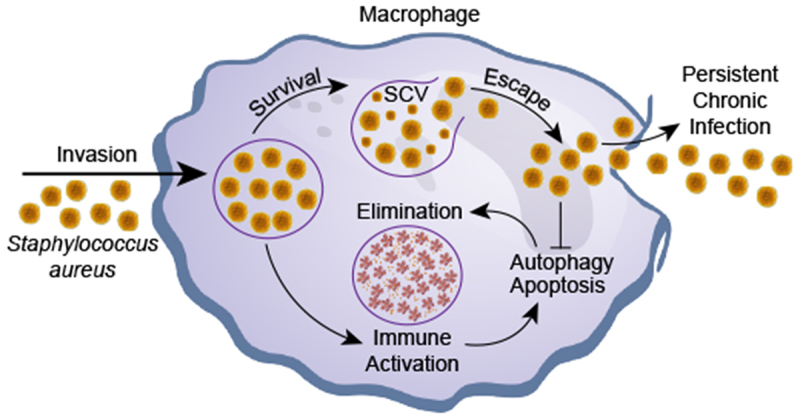

## The hidden life of *S.* *aureus*: from surface colonizer to intracellular survivor

When Alexander Ogston first described *Staphylococcus aureus* in the 1880s [1,2], he linked the bacterium to sepsis and abscess formation, laying the foundation for understanding its role in human disease. *S. aureus*, originally classified by its ability to ferment mannitol and produce coagulase, lipase, and a clumping factor [3], is a facultative anaerobic micrococcus colonizing about 30% of humans asymptomatically [4]. These colonization sites, primarily the anterior nares, skin, and gastrointestinal (GI) tract, serve as ecological niches influencing both transmission and infection risk [5]. While nasal carriage is the most studied and strongly associated with invasive disease and surgical site infections [6], skin colonization, especially at sites of barrier compromise, is increasingly recognized as a direct source of *S. aureus* in osteomyelitis [7,8]. GI colonization, though less understood, has also emerged as a potential reservoir [9].

Colonization is shaped by microbial competition, particularly with commensal *Staphylococcus epidermidis*, which produces antimicrobial peptides and quorum-sensing inhibitors that limit *S. aureus* growth [10,11]. Studies from Horswill and others have highlighted the roles of interspecies competition, biofilm formation, and the accessory gene regulator (Agr) quorum-sensing system in defining niche-specific colonization patterns [12–14]. Understanding these dynamics is essential for developing microbiome-targeted interventions aimed at decolonization and infection prevention.

Once skin or mucosal barriers are breached, *S. aureus* quickly establishes infection [15], particularly in skin and soft tissues, where it is often the first colonizer [16,17]. Immune evasion is tightly linked to Agr-mediated quorum sensing, which coordinates the expression of virulence factors in response to bacterial population density [18]. Activated Agr programs drive the production of toxins and adhesins, supporting both invasion and immune evasion [19]. Biofilm formation, initiated within hours of infection, further protects the bacteria by physically shielding them from immune recognition and impeding immune cell trafficking [20–23]. Biofilms are key to innate immune resistance and chronicity of infection. *S. aureus*-induced osteomyelitis, particularly following prosthetic joint replacements, differs significantly from other chronic infections (pulmonary or urinary tract) in its pathogenesis and treatment approach. In osteomyelitis, *S. aureus* forms robust biofilms on bone and implant surfaces, evading host immunity and antibiotics, necessitating physical debridement or prosthesis for treatment [24,25]. In contrast, *S. aureus* in chronic UTIs or pulmonary infections typically does not form complex biofilms and is often managed by antibiotic treatment [26,27]. These distinctions underscore the role of anatomical influence and foreign material in the persistence and treatment of *S. aureus* infections.

Macrophages are professional phagocytes that work alongside neutrophils to play a central role in the host response to *S. aureus* infection [28]. These tissue-resident phagocytes detect pathogens using pattern recognition receptors on their cell surface, which trigger cytokine and chemokine production to recruit additional immune cells and polarize circulating monocytes [29–31]. Once inflammatory monocytes traffic to the site of infection, they can further differentiate into macrophage or dendritic cell phenotypes to further contribute to generating a localized inflammatory response [32]. Upon bacterial encounter, macrophages engulf *S. aureus* and attempt to kill it using phagosomal acidification, reactive oxygen and nitrogen species, and degradative enzymes (e.g. proteases and lipases) [28]. However, *S. aureus* can persist intracellularly by escaping the phagosome or resisting lysosomal degradation [28,33]. Some strains impair autophagy [34], downregulate macrophage signaling [35], or modulate macrophage phenotype [36], all of which compromise clearance and promote intracellular survival.

Although most attention has focused on *S. aureus* as an extracellular pathogen forming abscesses and biofilms, mounting evidence supports a distinct intracellular lifestyle that contributes to chronic and relapsing disease. This is particularly relevant in osteomyelitis, a bone infection where macrophages and osteoclasts are frequently infected intracellularly [37]. Bone, often viewed as a protected site due to its mineralized matrix and low vascularity, becomes vulnerable following trauma, surgery, or vascular compromise [38]. Osteoblasts produce the extracellular bone matrix (osteoid), which is rich in collagens (up to 90%), glycoproteins, bone sialoprotein, osteocalcin, and essential for bone mineralization and development of mature bone tissue [39]. When damaged, the osteoid becomes a portal for bacterial entry. Bones with lower vascularization and softer matrix, such as the mandible, are more prone to infection [38].

Once *S. aureus* reaches bone, its intracellular survival within macrophages contributes to persistent inflammation and resistance to treatment [38,40]. The acute osteomyelitis environment is marked by hyper-inflammatory macrophages, many of which are infected, underscoring the need to understand how *S.* *aureus* exploits the intracellular niche to evade host defenses.

This review explores how *S. aureus* establishes intracellular infection within macrophages and how this contributes to osteomyelitis pathogenesis. We focus on the mechanisms of macrophage manipulation, immune evasion, and persistence in bone tissue, with the goal of highlighting potential therapeutic targets for treating chronic, hard-to-eradicate infections.

## Bone under siege: *S. aureus* and the pathogenesis of osteomyelitis

Osteomyelitis is a serious infection of bone tissue that can manifest as either acute or chronic disease. Despite its relative resistance to infection, bone can become vulnerable following trauma, surgery, vascular compromise, or the presence of implants [41]. These events can compromise the periosteum, which is the extracellular protective barrier that normally shields the bone marrow, allowing microbial access to the bone’s interior [8]. Bone is dense, mineralized tissue composed primarily of osteoblasts, osteoclasts, and osteocytes [39,42]. Osteoblasts are responsible for producing and secreting the extracellular bone matrix, driving bone formation [43]. Osteoclasts resorb old or damaged bone [42], clearing space for new tissue to be laid down. Osteocytes, which are embedded in the matrix, act as mechanosensors that regulate osteoblast and osteoclast activity [44]. Under healthy conditions, these cell populations work in balance to maintain skeletal integrity.

Although bone is poorly vascularized and therefore relatively protected from bloodstream infections, *S. aureus* has evolved strategies to overcome this barrier. The bacterium can exploit trauma or surgery to gain access to bone tissue and establish infection [38], especially in the presence of implanted devices, which impair immune surveillance and provide a surface for biofilm formation. Although all bone types are believed to be susceptible to infection [45], bones with low vascularization or great marrow exposure, such as the long bones or mandible, are especially vulnerable. Here, the extracellular protective barrier of the bone, or the periosteum, becomes damaged, allowing bacterial challenges to reach the bone marrow [39]. Risk factors for osteomyelitis include diabetes, immunosuppression, recent fractures, poor circulation, and intravenous drug use [46]. Diagnosis involves physical examination for fever, swelling, bony tenderness, or reduced range of motion [46], with imaging and biopsy used to confirm infection [46].

Once *S. aureus* enters bone tissue, it triggers a potent inflammatory response that recruits immune cells to the site of infection. Macrophages, in particular, are implicated in both the dissemination and persistence of *S. aureus* within bone [28,47]. These cells are among the first responders to bacterial challenge, but *S. aureus* can hijack them as intracellular reservoirs. Inside macrophages, the bacterium avoids clearance while stimulating the release of inflammatory cytokines and toxins, further damaging bone tissue and promoting osteomyelitis. In addition to serving as a bacterial reservoir, *S. aureus* has been observed to infect undifferentiated monocytes to stimulate further dissemination through the bloodstream [48].

Effective treatment of *S. aureus*-induced osteomyelitis is complicated by the bacterium’s intracellular lifestyle. Antibiotic selection must consider not only bone penetration but also intracellular activity. Drugs such as rifampin and clindamycin are effective against intracellular *S. aureus* within bone-resident cells, including osteocytes [47,49]. Complicating therapy further, some β-lactam antibiotics paradoxically activate the Agr system quorum-sensing system, increasing expression of cytotoxic exotoxins and exacerbating tissue damage [50]. In contrast, ribosomal inhibitors like clindamycin or linezolid are bacteriostatic but suppress Agr activity, which may mitigate immunopathology [51,52]. These findings highlight the importance of understanding how different antibiotic classes interact with *S. aureus* virulence regulation and intracellular persistence.

Virulence mechanisms that support intracellular survival are also tightly linked to *S. aureus* entry into bone tissue. The bacterium expresses adhesins such as fibronectin-binding proteins (FnBPs), which mediate attachment to host extracellular matrix proteins and facilitate invasion of host cells [53]. These adhesins target fibronectin, laminin, elastin, and bone sialoprotein [53], which are abundant matrix components and surface proteins in the osteoid, suggesting direct interactions between *S. aureus* and osteoblasts or osteocytes [54–56]. Though primarily characterized in vitro [56–58], these mechanisms likely contribute to cellular invasion in vivo. Small colony variants (SCVs) of *S. aureus*, which emerge during intracellular infection, are particularly adept at cell entry and immune evasion [59]. SCVs upregulate adhesins like FnBPs and polysaccharide intercellular adhesion proteins, enhancing their capacity for intracellular survival and persistence [60,61].

Beyond promoting cellular entry, *S. aureus* also directly damages bone tissue. Osteoclasts express receptors such as complement component 5a receptor, which mediate susceptibility to *S. aureus* pore-forming toxins like Panton-Valentine leukocidin [62]. These toxins destabilize osteoclast membranes and exacerbate bone resorption. At the same time, the infection shifts the balance of receptor activator of nuclear factor *κ*B ligand (RANKL)-mediated signaling and macrophage colony-stimulating factor, increasing osteoclast differentiation and activity [63–65], while inducing apoptosis in osteoblasts through tumor necrosis factor (TNF)-α and TRAIL (TNF-related apoptosis-inducing ligand). TRAIL can bind death receptor 4, osteoprotegerin, and death receptor 5, all of which are apoptosis promoting receptors [66]. *S. aureus* infection stimulates the secretion of TNF-α, which directly enhances the expression of RANKL [67], and when RANKL binds its receptor, RANK, osteoclast precursors begin to differentiate into mature osteoclasts to impact bone resorption [65]. *S. aureus* initiates the killing of osteoblasts by direct cell lysis from toxins (e.g. phenol soluble modulins (PSMs)) or induction of apoptosis [54,68], and the presence of apoptotic bodies within the bone can further stimulate inflammation and worsen the pathology [69]. This imbalance between osteoclasts and osteoblasts [68], or bone resorption and formation, leads to a decrease in bone density and progressive bone loss.

Cell death mechanisms beyond apoptosis also contribute to bone pathology during osteomyelitis. *S. aureus* infection induces pyroptosis in osteoblasts, mediated by proteins such as caspase-1, Nod-like receptor family 3 (NLRP3), and gasdermin D [70]. These markers are elevated in infected bone compared to healthy tissue, and inhibition of caspase-1 or NLRP3 in murine models of *S. aureus*-induced osteomyelitis alleviates bone damage and restores osteoblast function [70]. The resulting apoptotic and pyroptotic debris further fuels inflammation and osteoclastogenesis. Osteoclastogenesis defines the process by which osteoclast precursor cells differentiate to form mature, multinucleated osteoclasts [69].

Intracellular infection of macrophages also intersects with autophagy pathways. Autophagy is a conserved process that maintains macrophage homeostasis under stress, enabling the degradation of intracellular pathogens [71,72]. This process allows for the degradation and recycling of damaged cellular components, and in extreme cases, the initiation of apoptosis to prevent the remaining macrophage population from being exposed to the present stressor [72]. However, *S. aureus* can inhibit autophagic flux by Agr-mediated mechanisms, leading to bacterial accumulation within autophagosomes [73]. This not only permits bacterial persistence but also promotes inflammatory signaling that worsens the disease phenotype.

The inflammatory environment created by *S. aureus* in bone stimulates further osteoclastogenesis, in part through increased secretion of colony-stimulating factors from infected osteoblasts and macrophages [74]. Granulocyte colony-stimulating factor and granulocyte-macrophage colony-stimulating factor contribute to monocyte recruitment and osteoclast differentiation [74–76]. Once osteoclastogenesis is stimulated, osteoblasts increasingly interact with *S. aureus* and this process increases expression of monocyte chemoattractant protein-1 (MCP-1) and interleukin-6, sustaining monocyte recruitment and osteoclast activation [77,78]. These feedback loops reinforce the persistent inflammatory microenvironment and create a niche, within resident macrophages and osteoclasts, for *S. aureus* intracellular survival.

Taken together, these multifaceted interactions between *S. aureus*, immune cells, and bone-resident cells illustrate how osteomyelitis is not merely an infection of bone but a dynamic host-pathogen battle. Intracellular survival within macrophages and other cell types allows *S. aureus* to evade immune clearance and maintain an inflammatory state that both degrades bone and impairs healing. Understanding these mechanisms is essential for the development of targeted therapies that address not just the bacterial burden but also the dysregulated host response.

## The battle within: *S. aureus* manipulates host defenses for survival

The intracellular reservoir is particularly important in chronic infections, allowing *S. aureus* to evade immune clearance and persist despite antimicrobial therapy. During the early stages of infection, *S. aureus* interferes with host phagocytic mechanisms through secreted immune evasion factors. One such factor, extracellular fibrinogen binding protein (Efb), blocks recognition and engulfment by binding to fibrinogen, complement component C3b, and immunoglobulins, thereby inhibiting opsonization and phagocytosis [79,80].

Beyond immune evasion, *S. aureus* actively promotes cellular uptake, particularly into nonprofessional phagocytes such as osteoblasts, fibroblasts, and endothelial cells [53,81–84]. Tight bacterial adhesion is a prerequisite for internalization and is primarily mediated by fibronectin binding proteins (FnBPs) expressed on the bacterial surface [53]. These adhesins form a molecular bridge between bacterial cell wall components and host cell α5β1 integrin via fibronectin, initiating cytoskeletal rearrangements that drive bacterial engulfment [85,86]. The interaction is reinforced by tandem β zipper domains that cluster fibronectin molecules and enhance binding avidity [87]. While initially described in the context of endothelial and epithelial cells, this mechanism has been observed across a broad range of cell types, including osteoblasts and macrophages.

In addition to physical adhesion, *S. aureus* activates host signaling networks to promote internalization. These “invasion signaling” cascades are initiated by the recognition of staphylococcal ligands, including lipoproteins and other pathogen-associated molecular patterns, by pattern recognition receptors such as Toll-like receptor 2 (TLR2) [28,88,89]. TLR2 activation primes host innate immune cells by enhancing survival, cytokine production, chemokine secretion, and effector functions including phagocytosis and neutrophil exocytosis [90,91]. These responses are designed to coordinate antimicrobial defense but can be co-opted by *S. aureus* to facilitate its own uptake and intracellular survival.

Intracellular invasion by *S. aureus* also depends on modulation of host kinase signaling. In osteoblasts, successful uptake requires activation of mitogen-activated protein kinases (MAPKs) ERK and JNK, but not Elk-1 or ATF-2 [92,93]. Similarly, in endothelial cells, *S. aureus* internalization activates the phosphoinositide-3-kinase-protein kinase B pathway, which contributes to cytoskeletal remodeling and phagocytic uptake [94]. These findings indicate that *S. aureus* exploits both surface adhesion and intracellular signaling to invade host cells across tissue types.

Together, these host-pathogen interactions define an important intracellular phase of *S.* *aureus* infection. By circumventing immune recognition, leveraging host cell machinery for uptake, and exploiting immune signaling for its own benefit, *S. aureus* establishes a protected niche inside host cells.

## Macrophage hijacking: *S. aureus* in its macrophage hideout

Following internalization, *S. aureus* faces a range of possible intracellular fates, shaped by bacterial strain, virulence factor production, and host cell responses. For example, the USA300 clinical isolate displays the ability to survive and adapt to acidified phagosomal compartments, a trait that supports its intracellular persistence in immune cells [95,96]. Once inside the host cells, *S. aureus* may trigger either a robust cytotoxic response or remain metabolically quiescent, depending largely on the activation of its Agr quorum sensing system [85,97]. Agr activation drives expression of cytolytic toxins [98–101], including α- and β-hemolysins, which disrupt host membranes, β-toxin exhibits sphingomyelinase activity, degrading membrane lipids to destabilize the phagosome [102]. Host cells may counteract toxin activity through vesicular exocytosis [103], but this defense is often insufficient to eliminate infection.

The success of intracellular survival also depends on bacterial load and the physiological characteristics of *S. aureus* (e.g. SCV phenotype switching, virulence factor production) at the time of infection. At low multiplicity of infection (MOI) and during rapid bacterial growth, *S. aureus* is more likely to be killed through phagosome maturation and acidification [104]. This is evidenced by the degradation of green fluorescent protein-labeled *S. aureus* in neutrophils and primary macrophages [105], correlating with lysosomal fusion and myeloid differentiation primary response 88 (MyD88)-TLR signaling [106,107]. However, at higher MOI, macrophages may be overwhelmed, reducing their capacity for bactericidal activity [108,109].

Approximately 10% of internalized *S. aureus* survive phagolysosomal acidification through phenotypic switching into SCVs [110], a process supported by enzymes such as sortase A [111]. Sortase A, which remains active even in oxidizing lysosomal environments, catalyzes the anchoring of fibronectin-binding proteins and clumping factors, which are key adhesins that support intracellular persistence and biofilm formation [111,112]. SCVs exhibit reduced metabolism, slow growth, cytotoxicity, and downregulated *agr* expression, allowing them to evade immune surveillance [113]. These variants are highly persistent in host cells, capable of surviving for weeks and reactivating into virulent forms under permissive conditions, such as nutrient-rich environments or elevated pH [113]. Clinically, SCVs are associated with recurrent and treatment-refractory infections [85,113].

To further support survival, *S. aureus* produces PSMs, including PSMα, which permeabilize the phagosomal membrane, facilitating bacterial escape into the host cytoplasm [114]. The pathogen also induces LC3-associated phagocytosis, which delays phagosomal maturation and supports bacterial survival by mimicking autophagic compartments [115]. These mechanisms allow *S. aureus* to avoid degradation and persist intracellularly, creating a reservoir for dissemination to other tissues [73].

When host defenses fail, infected macrophages may initiate apoptosis as a last resort to limit bacterial spread [116]. However, *S. aureus* expresses an array of factors (Table 1), including hemolysins, leukocidins, and modulins, that can either accelerate or suppress apoptosis depending on the context [116,117]. In some cases, apoptosis is triggered prematurely to prevent immune activation; in others, the process is suppressed to promote bacterial survival. Suppression of apoptosis occurs by upregulation of anti-apoptotic proteins such as myeloid cell leukemia 1 (Mcl-1), mediated by nuclear factor *κ*B activation and IL-6 secretion [118]. In parallel, *S. aureus* inhibits cytochrome c release and caspase-3 activation, thereby stabilizing infected macrophages and prolonging the intracellular niche [118].

**Table 1.** *S. aureus* effectors in osteomyelitis.

*S. aureus* Effector Protein/Molecule	Function
Efb	Prevents bacterial recognition by binding fibrinogen, inhibiting phagocytosis and opsonization.
α-Toxin	Pore-forming toxin that creates holes in host cell membranes, leading to cell lysis.
β-Toxin	Breaks down lipids within the cell membrane, destabilizing the membrane and promoting cell death.
PSM-α	Disrupts the phagosomal membrane of macrophages prior to acidification, aiding in phagosomal escape and intracellular survival.
Sortase A	Bacterial enzyme that facilitates protein attachment on the cell wall, contributing to bacterial adherence, biofilm formation, and pathogenesis. It also aids in phenotypic switching to small colony variants (SCVs).
Leukocidins	Toxins that kill immune cells, including macrophages and neutrophils, impairing the host’s immune response.
Hemolysins	Exotoxins that lyse red blood cells and immune cells, contributing to tissue damage and immune evasion.
Clumping Factor	Contribute to bacterial adherence to host tissues and biofilm formation.
SpA	Surface protein that binds to immunoglobulin G (IgG), modulating immune responses and preventing phagocytosis and antibody-mediated recognition.
MIF	Stimulated by *S.* *aureus* protein A; contributes to immune cell recruitment, exacerbates pro-inflammatory responses, and promotes bone destruction.
MAPK	Signaling molecules that regulate immune responses and facilitate bacterial uptake into host cells (especially osteoblasts and endothelial cells).
Mcl1	Anti-apoptotic protein that inhibits macrophage apoptosis, allowing for bacterial survival and persistence.
IL-6	Cytokine involved in inflammation regulation; modulate macrophage responses and inhibit apoptosis.

This table summarizes key *S.* *aureus* proteins and molecules involved in immune evasion, intracellular survival, and bone destruction. These factors contribute to cytotoxicity, persistence, biofilm formation, and inflammation, underscoring the pathogen’s multifaceted strategies in sustaining osteomyelitis.

The apoptotic bodies generated during infection may contribute to local inflammation and osteoclastogenesis [119]. While typically cleared by phagocytic cells such as bone-resident macrophages, these apoptotic fragments can inhibit osteoblast differentiation and disrupt bone remodeling even if they are not a defining feature of osteomyelitis [119]. This disruption of normal bone cell turnover contributes to tissue degradation and failure of repair mechanisms. Ultimately, *S. aureus*’s ability to evade intracellular killing, manipulate host cell death pathways, and persist in quiescent forms underlies its capacity to cause long-term infection and drive chronic bone inflammation [26].

## Dual faces of macrophages: from fighters to facilitators

**Figure 1.**
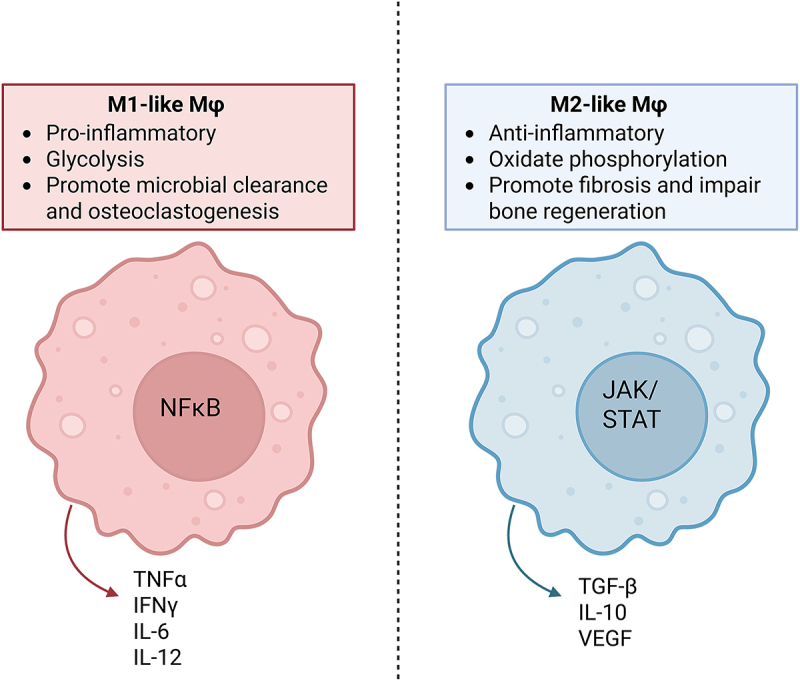
Role of macrophage phenotypes in osteomyelitis progression. The contrasting functions of M1- and M2-like macrophages in osteomyelitis are shown. M1-like macrophages (left, red) adopt a pro-inflammatory, NF-κB – driven profile, producing cytokines like IL-6, relying on glycolysis, promoting microbial clearance, and enhancing osteoclastogenesis and bone resorption. M2-like macrophages (right, blue) exhibit an anti-inflammatory, JAK/STAT – mediated phenotype, secrete factors like VEGF, use oxidative phosphorylation, and promote fibrosis while impairing bone regeneration. The balance between these phenotypes’ shapes disease severity and chronicity. Figure generated using BioRender.

Macrophages are highly plastic cells that can adopt distinct functional phenotypes in response to environmental cues, including microbial signals, cytokines, and metabolic stressors (Figure 1). This plasticity enables them to transition from immune defenders to contributors of persistent infection [120]. Monocyte differentiation into bone resident macrophages is driven primarily by M-CSF stimulation [121]. This process occurs over the course of 3–7 days, allowing for circulating monocytes to traffic and differentiate at the site of infection [121,122]. During the early stages of *S. aureus* osteomyelitis, monocytes infiltrating bone tissue are exposed to a surge of pro-inflammatory cytokines, which drives them toward a classically activated, pro-inflammatory state [123]. These M1-like macrophages exhibit increased chemotaxis, phagocytosis, and production of reactive oxygen and nitrogen species to combat bacterial infection. However, *S. aureus* infection also reprograms macrophage metabolism and signaling pathways, progressively skewing polarization toward an M2-like phenotype. Although originally described using a binary M1:M2 framework [121,124], macrophage phenotypes exist along a spectrum. The M1-like phenotype is associated with glycolytic metabolism, antimicrobial activity, and high expression of cytokines such as TNF-α and IL-12 [125]. In contrast, M2-like macrophages rely on oxidative metabolism and promote tissue repair and immune resolution. Subtypes of M2 macrophages (M2a, M2b, M2c, M2d) differ in surface markers and secretory profiles but all share the expression of IL-10 and contribute to anti-inflammatory functions [126]. While this dichotomy is useful for conceptualizing macrophage function, in vivo macrophage populations are far more heterogeneous and context-dependent [127]. Considering this complexity, an alternative system for classifying macrophages has been proposed based on their activating ligands or stimuli instead of polarization state [127].

In acute bacterial infections, a typical trajectory involves an early M1 response to eliminate the pathogen, followed by a shift toward M2 polarization to promote tissue regeneration. In chronic *S. aureus* osteomyelitis, however, this transition is dysregulated [128,129]. Prolonged infection and persistent inflammation disturb the balance between M1 and M2 macrophage populations, resulting in a skewed M1:M2 ratio that fails to resolve inflammation or repair damaged bone [120]. In some contexts, an overabundance of M1 macrophages exacerbated tissue damage, while premature or misdirected M2 polarization facilitates bacterial persistence and immune evasion [129].

This phenotypic shift is orchestrated by signaling pathways including STAT1, which promotes M1 polarization, and STAT3/STAT6, which drive M2-like polarization [128]. *S. aureus* exploits these pathways to manipulate macrophage behavior. For example, intracellular infection triggers STAT3/STAT6 signaling and IL-10 production, which suppress pro-inflammatory functions and support bacterial persistence [128,130]. Moreover, biofilm-associated *S. aureus* has been shown to further modulate macrophage metabolism. In the absence of biofilm, macrophages rely on glycolysis and maintain an M1-like profile [131]; however, in the biofilm microenvironment, sequestration of glucose and other nutrients leads to a metabolic shift favoring oxidative phosphorylation and M2 polarization [132].

Thus, macrophage polarization is a double-edged sword: while essential for both bacterial clearance and tissue repair, it can be co-opted by *S. aureus* to promote chronicity. An inappropriate or premature shift toward M2 macrophages results in impaired pathogen clearance and ongoing immune suppression, creating a permissive niche for *S. aureus* survival. The dynamic interplay between macrophage phenotype and infection stage highlights the importance of temporarily regulated immune responses in controlling osteomyelitis.

## Macrophages in the crossfire: amplifiers of bone damage

Beyond modulating macrophage polarization, *S. aureus* infection impairs the functional capacity of bone-resident macrophages to mediate bone remodeling during osteomyelitis [133]. As first responders to infection, macrophages secrete chemokines that recruit additional immune cells and initiate tissue-level responses. Chemokines such as CXCL2 and CCL4 attract circulating monocytes and neutrophils while simultaneously promoting osteoclast differentiation [134]. Other chemokines produced by resident macrophages, including CXCL8, CXCL10, and CXCL20, further support osteoclastogenesis and influence repair processes [134].

A key immune modulator in this process is macrophage migration inhibitory factor (MIF), which is upregulated in response to staphylococcal protein A [135]. MIF is a pleiotropic chemokine-like cytokine produced by multiple immune cell types, originally identified as an inhibitor of glucocorticoid suppression of inflammation [136]. In the context of *S. aureus* osteomyelitis, MIF amplifies pro-inflammatory signaling, recruits additional macrophages to infected bone, and directly contributes to bone destruction by interfering with osteogenic differentiation [135]. As MIF expression increases, the inflammatory loop intensifies, driving further macrophage infiltration and sustaining tissue damage.

In parallel, *S. aureus* actively interferes with macrophage recognition, phagosome-lysosome fusion, pH regulation, and membrane integrity to block bacterial clearance [28,137]. Toxins secreted by *S. aureus* disrupt membrane stability and diminish macrophage phagocytic function. Meanwhile, biofilm formation contributes significantly to immune evasion. Biofilms trap and deactivate macrophages at the infection site, shielding *S. aureus* from immune recognition and antimicrobial treatments [138]. These bacterial communities, embedded in a self-produced matrix, are frequently found within necrotic bone and can adhere directly to bone surfaces [139]. While biofilms are commonly associated with implanted devices, they can also form in native bone, particularly in chronic or device-associated osteomyelitis [139,140]. Device-associated biofilms often act as reservoirs, seeding recurrent infection and exacerbating disease progression.

**Figure 2.**
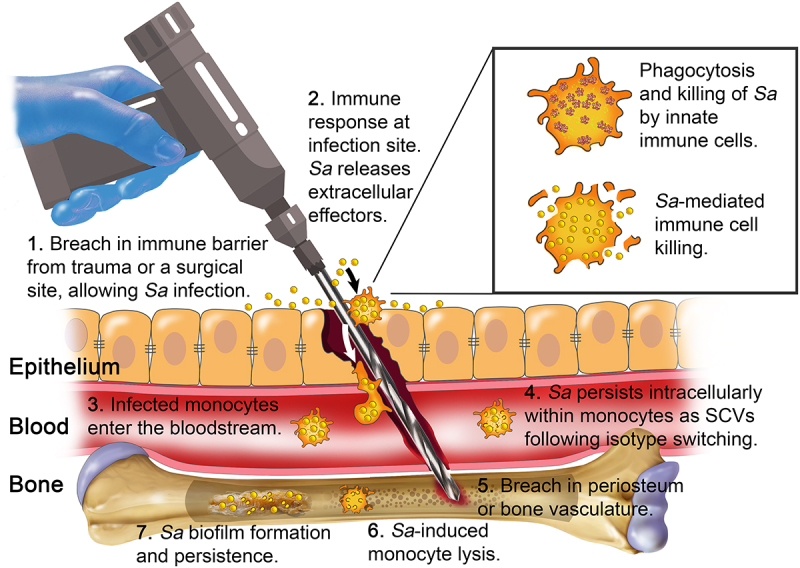
*S. aureus* infection pathway to bone. This flow diagram outlines the progression of *S. aureus* (*Sa*) infection leading to osteomyelitis. Infection begins with a breach in host barriers, followed by tissue invasion and entry into the bloodstream (bacteremia). Upon reaching bone, *S. aureus* adheres to the matrix, colonizes the site, and triggers an immune response. Bacterial survival is aided by immune evasion strategies, including intracellular persistence within macrophages and phenotype switching to small-colony variants (SCVs). These adaptations promote antibiotic resistance and allow repeated cycles of escape and recolonization, resulting in bone destruction, abscess formation, and chronic infection.

Macrophage polarization during infection has functional consequences for osteoclastogenesis and bone remodeling. Two key principles have emerged [37] (1) M2-like macrophages promote both bone repair and osteoclast differentiation, and (2) M0- or M1-like macrophages inhibit osteoclast formation while favoring new bone deposition [141]. *S. aureus* exploits these principles by perturbing the natural progression of macrophage polarization. In an effective immune response, infection induces M1-like macrophages to clear bacteria, followed by a switch to M2-like macrophages to initiate healing [142]. However, in persistent infections, *S. aureus* alters this trajectory and migrates through infected monocytes (Figure 2). By driving a premature or dysregulated M2-like phenotype, the bacteria avoid immune clearance and disseminate to secondary sites [143].

Evidence suggests that this M1-to-M2 oscillation continues at each new infection site [144]. In the bone, high bacterial density triggers a rebound in M1-like polarization, augmenting inflammation and cytokine release [143]. This is reminiscent of macrophage behavior in other inflammatory bone diseases, such as osteoporosis and osteoarthritis, where an elevated M1:M2 ratio contributes to chronic inflammation and tissue damage [145]. In osteomyelitis, an overrepresentation of M1-like macrophages leads to excessive production of pro-inflammatory cytokines and reactive oxygen species, which further degrade bone [146]. Simultaneously, the depletion of M2-like macrophages reduces vascularization and impairs bone regeneration [147]. The net effect is a sustained inflammatory environment in which the destructive capacity of M1 macrophages exceeds the reparative capacity of M2 macrophages, perpetuating bone loss and disease chronicity.

## New frontiers in osteomyelitis: targeting macrophage polarization

The ability of *S. aureus* to manipulate macrophage polarization is a central factor in the persistence and chronicity of osteomyelitis. While traditionally viewed as an extracellular pathogen, *S. aureus* displays a sophisticated intracellular lifestyle that allows it to evade host defenses and antimicrobial therapy. Once engulfed, *S. aureus* can survive within macrophages, establishing a protected niche that facilitates dissemination and immune evasion. This intracellular persistence presents a major challenge to both innate immune responses and clinical treatment strategies.

The initial immune response to *S. aureus* infection typically involves a shift toward M1-like macrophage polarization. These macrophages produce pro-inflammatory cytokines and reactive oxygen species and engage in phagocytic killing to eliminate bacteria and initiate tissue repair. However, *S. aureus* subverts this host response by triggering a transition to the M2-like macrophage phenotype. M2-like macrophages, while essential for resolution of inflammation and tissue repair in noninfectious contexts, become maladaptive during infection. Their anti-inflammatory and immunosuppressive profile dampens host defense mechanisms, allowing *S. aureus* to persist and propagate within the infected tissue.

Despite these challenges, the dynamic interplay between *S. aureus* and macrophage polarization offers promising therapeutic opportunities. Strategies that enhance M1-like activity or prevent premature M2-like polarization could restore antimicrobial effector functions and improve bacterial clearance. Conversely, modulating the inflammatory response to avoid excessive bond destruction, while maintaining bacterial killing, could help balance tissue repair with host defense. Pharmacologic inhibition of key bacterial factors such as Agr-related toxins, SCV formation, or biofilm maturation may synergize with host-directed therapies to limit intracellular survival and reduce chronicity.

A deeper understanding of macrophage plasticity in response to bacterial cues will be essential for developing next-generation therapies for osteomyelitis. Targeting the molecular pathways that control macrophage polarization, including the STAT, NF-*κ*B, and metabolic regulators, may provide a framework for tipping the balance in favor of resolution. Therapies aimed at disrupting the intracellular lifestyle of *S. aureus* or restoring autophagic flux within infected macrophages may also prove beneficial in eradicating hidden reservoirs of infection.

In summary, macrophage polarization represents a key axis of *S. aureus* pathogenesis in osteomyelitis. By elucidating and manipulating the molecular dialogue between pathogen and host immune cells, researchers can uncover novel strategies for treating persistent bone infections. These insights may ultimately extend beyond osteomyelitis, offering broader implications for managing chronic infections in which bacterial persistence and immune modulation converge.

The abbreviations used are as follows: *agr*, accessory gene regulator; FnBP, fibronectin binding proteins; SCV, small colony variants; TRAIL, TNF-related apoptosis-inducing ligand; RANKL, receptor activator of nuclear factor *κ*B ligand; IL, interleukin; Mcl1, myeloid cell leukemia 1; STAT, signal transducer and activator of transcription proteins; MIF, migration inhibitory factor; MOI, multiplicity of infection; myeloid differentiation primary response 88, MyD88; Toll-like receptor, TLR; MAPK, mitogen-activated protein kinases; M1, classically activated macrophages; M2, alternatively activated macrophages

## Data Availability

Data sharing is not applicable to this article as no new data were created or analyzed in this study.
